# Evaluation of a competitive hepcidin ELISA assay in the differential diagnosis of iron deficiency anaemia with concurrent inflammation and anaemia of inflammation in elderly patients

**DOI:** 10.1186/s12950-017-0166-3

**Published:** 2017-09-18

**Authors:** Torbjörn Karlsson

**Affiliations:** Department of Haematology, Uppsala University Hospital, 751 85 Uppsala, Sweden

**Keywords:** Hepcidin, Elisa, Anaemia, Iron deficiency, Inflammation

## Abstract

In this study, a competitive hepcidin ELISA assay was evaluated for its ability to differentiate between iron deficiency anaemia with concurrent inflammation and anaemia of inflammation in elderly patients, using the absence of stainable bone marrow iron as the diagnostic criterion for iron deficiency. In addition, correlation coefficients for hepcidin versus C-reactive protein, ferritin and interleukin-6 were determined. The optimal cut-off for hepcidin was 21 μg/L, corresponding to sensitivity and specificity of 100% and 67%, respectively, for iron deficiency. For ferritin, a sensitivity and specificity of 91% and 83%, respectively, correspond to an optimal cut-off of 87 μg/L. Receiver operating characteristics curve analysis revealed that ELISA analysis of hepcidin is not superior to ferritin in the diagnosis of iron deficiency in elderly anaemic patients with concurrent inflammation. Hepcidin shows a strong positive correlation with ferritin, and also correlates positively with C-reactive protein in this patient population.

## Introduction

Anaemia in elderly patients is commonly caused by iron deficiency or inflammation [1]. In this category of patients, iron deficiency is often difficult to diagnose biochemically, since an inflammatory response induced by, for example cancer, infection or autoimmune disease influences the levels of iron, transferrin and ferritin [2]. In anaemia of inflammation bone marrow aspiration and iron staining can be used as a supplementary diagnostic feature [3–5]. The hepatic bactericidal protein hepcidin has a central role in iron metabolism, and the molecular mechanisms behind hepcidin-induced ferroportin degradation, iron sequestration in the reticuloendothelial system, hypoferremia, and iron-restricted erythropoiesis in anaemia of inflammation (AI) have been elucidated in recent years [6]. Besides the biologically active hepcidin-25, human hepcidin is present in two other isoforms with molecular weights of 22 and 20 kDa, respectively. Hepcidin can be determined by enzyme immunoassay and mass spectrometry [7–9], and in iron deficiency anaemia (IDA), hepcidin is low or undetectable, whereas the level is normal or elevated in AI [7–9]. In this study, a competitive hepcidin ELISA assay was evaluated for its ability to differentiate between IDA with concurrent inflammation and AI in elderly patients. It would be a great advantage if an ELISA assay could be used for this purpose, since mass spectrometry analysis is considerably more labour intensive and expensive than ELISA analysis. This is, to our knowledge, the first study reported that use the lack of stainable bone marrow iron as the diagnostic criterion for iron deficiency (ID) in combination with a hepcidin ELISA assay in this patient category.

## Patients and methods

This study was performed at the Department of Haematology, Uppsala University Hospital, Uppsala, Sweden. Thirty consecutive patients newly diagnosed with anaemia (Hb <130 g/L in men and <120 g/L in women) with C-reactive protein (CRP) > 5 mg/L and 65 years of age or older were included. Except for the hepcidin and interleukin-6 analyses all tests were part of routine anaemia evaluation. Patients who had received iron supplementation or red blood cell transfusions within 1 month before testing were excluded from the study, as were patients with B12 or folate deficiency, haemolytic anaemia or any haematologic malignancy. Bone marrow smears were stained using the May-Grünwald-Giemsa method, and bone marrow iron stores were investigated using Prussian blue staining. Patients with no stainable bone marrow iron (*n* = 10) were diagnosed as having IDA with concurrent inflammation (ID-AI group). The diagnosis for those with stainable iron (*n* = 20) was AI. All analyses, with the exception of bone marrow, hepcidin and interleukin-6 analyses, were performed at the Department of Clinical Chemistry, Uppsala University Hospital. The CELL-DYN Sapphire Hematology System (Abbot Diagnostics, Lake Forest, IL, USA) was used to determine Hb, MCH and MCV. Iron, ferritin, transferrin and CRP were analysed using ARCHITECT Plus *ci* 16,200 (Abbot Diagnostics, Lake Forest, IL, USA). Transferrin saturation (TSAT) was calculated using the formula: TSAT (%) = (iron [μmol/L] / (transferrin [g/L] * 25.1)) * 100. The bone marrow analyses were performed at the Department of Pathology, Uppsala University Hospital. Patient sera for hepcidin and Interleukin-6 (IL-6) analyses were stored at −70 C before analysis. Hepcidin and IL-6 analyses were performed at the Study Center for Laboratory Medicine, Karolinska Hospital, Solna, Sweden. A commercially available competitive ELISA kit (Peninsula Laboratories International, San Carlos, CA, USA) was used for hepcidin analysis. IL-6 was analysed using a sandwich ELISA kit (Roche Diagnostics, Sweden). Detection range for this IL-6 assay is 1.5–5000 ng/L. Laboratory reference values for MCV are 82.0–98.0 fL, MCH 27–33 pg, iron 9–34 μmol/L, transferrin 1.94–3.26 g/L, TSAT 15–60% and 15–50% for men and women, respectively. The reference values for ferritin is 25–310 μg/L for men and 15–150 μg/l for women, for reticulocytes 26–130 * 10^9^/L and for CRP < 5 mg/L and Interleukin-6 < 7.0 ng/L. For hepcidin the references values are 2–31 and 8–76 μg/L for women and men, respectively. Statistical and receiver operating characteristic (ROC) curve analyses were performed using the SigmaPlot 11 software package (Systat Software, San Jose, CA, USA). This software program uses the Kolgomorov-Smirnov test for evaluation of data distribution. Depending on the distribution of data, the Student’s *t*-test or Mann-Whitney rank sum test was used to compare variables. Correlation coefficients were calculated using Pearson product moment correlation test. A *p*-value less than 0.05 was considered statistically significant.

## Results

Nine out of 10 (90%) of the iron-deplete patients with concurrent inflammation suffered from gastrointestinal haemorrhage, whereas the most common diagnoses in the population with AI were infection and autoimmune disease, that is, rheumatoid arthritis and giant cell arteriitis (Table 1). Mean age, iron, reticulocytes and IL-6 did not differ significantly between the ID-AI and AI population. As expected, mean MCV, MCH, TSAT and ferritin levels were significantly lower in the ID-AI group (*p* < 0.01, < 0.05, < 0.01 and <0.001, respectively), whereas mean Hb, CRP and hepcidin was higher in the AI population (*p* < 0.05, < 0.05 and <0.01, respectively). Mean hepcidin was 8.5 and 44 μg/L in the ID-AI and AI groups, respectively. Patient characteristics are shown in Table 2. Hepcidin correlated positively with CRP and ferritin (Table 3). There was a weak positive correlation between hepcidin and IL-6. ROC curve analysis revealed that the optimal cut-off for hepcidin was 21 μg/L, corresponding to a sensitivity of 100% and a specificity of 67%, respectively, for ID (Table 4). For ferritin, a sensitivity of 91% and a specificity of 83%, respectively, correspond to an optimal cut-off of 87 μg/L (Table 4). Area under the curve for ROC (AUC^ROC^) did not differ significantly between hepcidin (0.89) and ferritin (0.93). AUC^ROC^ for iron (iron vs. hepcidin *p =* 0.03) was significantly smaller than AUC^ROC^ for hepcidin (Table 4), whereas AUC^ROC^ for MCH and TSAT, respectively, was not (MCH vs. hepcidin, *p* = 0.22; TSAT vs. hepcidin, *p* = 0.11) (Table 4). Sensitivity, specificity and optimal cut-offs for ID, ROC area data and predictive values for MCH, iron, TSAT, ferritin and hepcidin are presented in Table 4.

**Table 1:** Clinical diagnosis according to type of anaemia

Clinical diagnosis	IDA-AI	AI
Gastrointestinal-haemorrhage	9	0
Malabsorption	1	0
Infection	0	5
Autoimmune disease	0	6
Unexplained anaemia	0	5
Miscellaneous	0	4

*IDA-AI* iron deficiency anaemia with concurrent inflammation, *AI* anaemia of inflammation

**Table 2:** Patient characteristics according to type of anaemia

Variable	Reference value	IDA-AI	AI
Age (years)	–	74 (70–92)	78 (69–88)
Hb (g/L)	>130 men, >120 women	103 (86–115)	113 (93–125)
MCV (fL)	82.0–98.0	87.7 (63.1–92.3)	93.1 (84.0–99.3)
MCH (pg)	27–33	28 (20–32)	31 (27–34)
Iron (μg/L)	9–34	6 (2–14)	9 (2–16)
Transferrin (g/L)	1.94–3.26	2.77 (1.71–4.03)	1.79 (1.27–2.64)
TSAT (%)	15–60 men, 15–50 women	9 (2–27)	18 (5–34)
Ferritin (μg/L)	25–310 men, 15–150 women	18 (10–95)	260 (32–1685)
CRP (mg/L)	<5	6 (6–44)	24 (6–154)
IL-6 (ng/L)	<7.0	3.3 (1.5–20)	8.0 (1.5–169)
Hepcidin (μg/L)	8–76 men, 2–31 women	4 (1–20)	35 (10–115)
Reticulocytes (×10^9^/L)	26–130	74 (29–145)	47 (24–117)

*IDA-AI* iron deficiency anaemia with concurrent inflammation, *AI* anaemia of inflammation, *Hb* haemoglobin, *MCV* mean corpuscular volume, *MCH* mean corpuscular haemoglobin, *TSAT* transferrin saturation, *CRP* C-reactive protein, *IL-6* interleukin-6

Data are presented as median (range)

**Table 3:** Correlation coefficients (Pearson product moment correlation) of hepcidin with Hb, CRP, iron, ferritin and IL-6

Variable	r (variable vs hepcidin)	*p*
Hb	−0.14	0.45
CRP	0.62	< 0.001
iron	−0.24	0.22
ferritin	0.85	<0.001
IL-6	0.49	0.01

*CRP* C-reactive protein, *IL-6* interleukin-6

**Table 4:** Test characteristics based on optimal cut-off values for iron deficiency, determined by ROC curve analysis and comparison between AUC^ROC^ for MCH, Iron, TSAT and ferritin, respectively, and AUC^ROC^ for hepcidin

Variable	Sensitivity (%)	Specificity (%)	ROC area	Cut-off	PV+ (%)	PV- (%)	*p*
MCH	56	89	0.78	28 pg	83	67	0.22
Iron	73	74	0.63	6.5 μmol/L	73	73	0.03
TSAT	70	74	0.74	14.5%	73	71	0.11
Ferritin	91	83	0.93	87 μg/L	84	90	0.58
Hepcidin	100	67	0.90	21 μg/L	75	100	–

*ROC* receiver operating characteristics, *MCH* mean corpuscular haemoglobin, *TSAT* transferrin saturation, *PV+* positive predictive value, *PV* negative predictive value

## Discussion

The aim of this study was to evaluate a competitive hepcidin ELISA assay in the differential diagnosis of IDA with concurrent inflammation and AI in elderly patients. The absence of stainable bone marrow iron was used as the diagnostic criterion for ID. As in our previous studies, gastrointestinal haemorrhage was the dominant diagnosis in the IDA group, whereas infection and autoimmune diseases (i. e. rheumatoid arthritis and giant cell arteriitis) were common diagnoses in the AI group [10, 11]. Five out of 30 (17%) of the patients in the AI population suffered from anaemia without any identifiable underlying pathologic condition. Unexplained anaemia (UA) is common in the elderly; for example, in the National Health and Nutrition Examination Survey III approximately 30% of the anaemic subjects had anaemia of such type [12]. Given the high prevalence of UA in the elderly a lower cut off to define anemia and for initiation of anaemia evaluation in geriatric subjects has been proposed [13, 14], but in this study we have used the haemoglobin cut-offs defined by the Department of Clinical Chemistry at our Hospital.

Soluble transferrin receptor-ferritin index (TfR-F index) and the Thomas plot have high sensitivity and specificity for ID in anaemic patients with concomitant inflammation [15, 16], and bone marrow aspiration with subsequent iron staining can be used as a supplementary diagnostic feature for ID during inflammation [3–5]. Currently, the best way to identify iron deficiency during concurrent inflammation is probably to use the TfR-F index, that is, sTfR/log ferritin. In a study by Punnonen and co-workers [15] AUC^ROC^ for this index was 1.0 when identifying anaemic patients with depleted iron stores. This index is also superior to analysis of ferritin alone in the diagnosis of iron depletion in patients with inflammatory bowel disease [17] and in the prediction of responsiveness to intravenous iron supplementation in patients with chronic kidney disease [18]. In addition, in a prospective multicenter evaluation of the TfR-F index it was shown that this ratio was superior to both analysis of ferritin and of soluble transferrin receptor in the diagnosis of iron depletion in patients with concurrent AI [19]. Since we did not analyse soluble transferrin receptor in this study, we used bone marrow aspiration as a supplementary diagnostic feature. The importance of using this criterion for ID in our patient population is evident when considering that the mean ferritin in the ID-AI population was 37 μg/L. If the conventional ferritin cut-offs for ID of 15 and 25 μg/L for women and men, respectively, had been used, 80% (8/10) of the iron-deficient patients in this study would have been misdiagnosed as iron replete. A ferritin cut-off for ID of 30 μg/L is commonly used in different clinical situations and in research [20–22] but even if this ferritin cut-off had been used 60% of the patients with ID in the present study would have been classified as iron replete. The optimal ferritin cut-off for ID in this study was 87 μg/L. This cut-off value is approximately two times higher than we observed in a previous study with a similar design [10, 11] and that others have observed [15, 23]. The reason for this difference is not known. Mass spectrometry analysis of hepcidin is specific for biologically active hepcidin-25, and differentiates between patients with IDA and AI [24, 25]. In contrast to the ELISA developed by Butterfield and co-workers. [26], the competitive ELISA assay used in this study is not specific for hepcidin-25 [27], but it cross-reacts with hepcidin-20 and -22. However, hepcidin levels determined by this assay correlated significantly with hepcidin-25 determined by mass spectrometry in the study by Dahlfors and co-workers [27]. Due to cross-reactivity with hepcidin-20 and -22, the level of hepcidin was approximately 30% higher when hepcidin was analysed using the ELISA assay compared to the level using hepcidin-25 specific mass spectrometry [27]. Although not specific for hepcidin-25, the mean hepcidin level was significantly lower in subjects with ID compared to healthy controls using this ELISA assay [27]. In the study reported here, we show a significant difference in hepcidin levels when comparing patients with ID-AI to those with AI. The mean hepcidin levels were 8.5 and 44 μg/L for the ID-AI and AI populations, respectively. The mean hepcidin was higher in the ID-AI population than that in the iron-deplete subjects in the Dahlfors study [27], probably because the iron-deplete patients in our study suffered from concomitant inflammation. The problem of identifying an underlying ID in the context of AI has been addressed in several populations, using ELISA assays. For example, the first commercially available hepcidin ELISA assay did not differentiate between IDA and AI in geriatric patients [28]. To our knowledge, only three other such published studies addressing this problem have used the absence of stainable bone marrow iron as the diagnostic criterion for ID [29–31]. In two of these studies hepcidin levels were considerably higher in all patient groups than we observe in this report. In addition, Shu et al. (30) reported an optimal hepcidin cut-off for ID of 83 μg/L, approximately four times higher than the optimal cut-off of 21 μg/L reported in this study. In a recent study using mass spectrometry-based hepcidin analysis we showed an optimal hepcidin cut-off for ID of 31 μg/L in a similar patient population with ID-AI or AI (11). One possible explanation for this difference is that another ELISA kit (Sandwich ELISA Uscn Life Science; hepcidin antibody E1979HU; mean hepcidin concentration 79 μg/L in controls) was used [29, 30]. In the third study, Barsan and co-workers used the same hepcidin ELISA kit as we did. They did not find that hepcidin was superior to ferritin analysis in its ability to differentiate between iron deficiency anaemia and anaemia of inflammation in patients with chronic kidney disease [31]. This is in agreement with our results reported here. However, in that report [31] the optimal hepcidin cut-off for ID was 82 μg/L. This discrepancy can at least to some extent be explained by the fact that hepcidin is excreted in the urine, and that patients with renal failure have higher levels of hepcidin in serum than healthy controls [9]. Applying a hepcidin cut-off of approximately 80 μg/L on our patient population would yield a much lower specificity for ID and a lower Yuoden Index. Sensitivity and specificity data for ID for different hepcidin cut-offs are presented in Table 5. We also confirm the positive correlation between expression of hepcidin and ferritin that we and others have described previously [9, 11, 29]. IL-6 is the main positive regulator of hepcidin expression [32], but we did not find a significant difference in mean IL-6 levels between patients with ID-AI and AI and only a weak positive correlation between hepcidin and IL-6. This indicates that other pro-inflammatory cytokines are involved in the regulation of hepcidin, since we did detect a significant difference in CRP between the two groups. It has been shown previously that increased erythropoiesis rather than serum iron negatively regulates hepcidin expression [33, 34]. Since we could not detect any differences in iron or reticulocyte count between the ID-AI and AI groups, we suggest that hepcidin is mainly regulated positively by iron stores and inflammation in the patient population reported here. We have previously shown that hepcidin analysis by mass spectrometry does not appear to be superior to ferritin in the differential diagnosis of ID with concurrent inflammation and AI in elderly patients [11]. In conclusion, in the present report we show that hepcidin analysed by a competitive ELISA is not superior to ferritin, using a modified optimal ferritin cut-off, in detecting iron deficiency in this patient category.

**Table 5:** Sensitivity, specificity, predictive values and Yuoden Indices for different hepcidin cut-offs for iron deficiency

Hepcidin cut-off (μg/L)	Sensitivity (%)	Specificity (%)	PV+ (%)	PV- (%)	Yuoden Index
18.5	82	78	79	81	0.60
19.5	81	67	71	79	0.48
21.0	100	67	75	100	0.67
24.5	100	56	69	100	0.56
35.0	100	50	67	100	0.50
76.5	100	17	55	100	0.17
89.0	100	11	53	100	0.11

*PV+* positive predictive value, *PV-* negative predictive value

## Abbreviations

AI: anaemia of inflammation
AUC^ROC^: area under the curve for ROC
CRP: C-reactive protein
Hb: haemoglobin
ID: iron deficiency
IDA: iron deficiency anaemia
IL-6: interleukin-6
MCH: mean corpuscular haemoglobin
MCV: mean corpuscular volume
ROC: receiver operating characteristic
TSAT: transferrin saturation
